# The principles of systems biology

**DOI:** 10.1113/EP093268

**Published:** 2026-03-26

**Authors:** Denis Noble, Reine Bourret

**Affiliations:** ^1^ Department of Physiology, Anatomy & Genetics University of Oxford Oxford UK; ^2^ Independent researcher Paris France

**Keywords:** biological relativity, central dogma, multi‐scale causation, systems biology

## Abstract

Physiological interpretations of Systems Biology have made many advances since the Principles of Systems Biology were first published in this journal in 2008. Those advances show that the main principle, Biological Relativity, is a logical necessity since no system can exist without the form of the system. That form creates the necessary boundary conditions for the integration of any equations for the mechanics by which the elements of the system interact. A further conclusion is that the Central Dogma of molecular biology is an incomplete representation of causation in biology. It neglects the multi‐scale properties of living systems, and its assumption that DNA can self‐replicate accurately enough is incorrect. The replicator cannot therefore be separate from its vehicle, the living cell. This failure has led to a gene‐centric impasse: the failure of genomics to produce the expected cures for common fatal diseases. Physiology now needs to provide alternative ways in which this problem can be successfully addressed.

## Abstract (highlights)

- **What is the topic of this review?**
  This review sets out the principles of systems biology.
- **What advances does it highlight?**
  This review includes fundamental corrections of gene‐centric biology that lead to understanding why the principles of Systems Biology are necessary.

## 1 INTRODUCTION

It is nearly two decades since the Principles of Systems Biology were formulated in this journal as a tribute to the 19th Century systems biologist, Claude Bernard (Noble, 2008a). That article has accumulated over 300 citations in fields as widespread as mathematics, physics, engineering, computer science, social sciences and philosophy. It is particularly important to note the inclusion of engineering science, mathematics and computer science. The two authors of this article extending the Principles have therefore collaborated in the work involved. We are both physiologists in origin, having interacted in Poitiers years ago. But one of us (R.B.) branched off into complex engineering in the field of aeronautics where she even became a lead pilot for a well‐known international airline. Our article will therefore draw on insights from Systems Engineering as well as Systems Biology, and it will be restricted to the physical levels of interaction in living organisms, i.e. principles 1–8 in the 2008 article.

## 2 PRINCIPLE 1: BIOLOGICAL FUNCTION IS MULTI‐LEVEL

When first formulated (Noble, 2008a), this principle expressed the fundamental nature of physiology, which has always been a multi‐level systems science, since a level in any complex system corresponds to similarity in complexity, dynamic properties and behaviour. It is not an accident that physiologists refer to the *systems* of the body. Nor that mathematics is involved. Claude Bernard expressed the need for mathematics in physiology (Bernard, 1865), just as in engineering. Engineering deals with either static states (buildings, bridges, etc.) or dynamic states (hydraulics, electrical, mechanical systems and feedback processes), just as systems biology must address quantitatively both form and mechanics (anatomy, development and physiology). Multiple levels of analysis are also deep in the historical origins of physiology, defined by Jean Fernal (*De naturali parte medicinae*) in 1542 (Sherrington, 1946).

This long‐standing feature of physiology was dismissed by the rise of molecular biology, the central tenet of which was Crick's Central Dogma (Crick, 1958, 1970). Not only did that dogma challenge the validity of any systems‐based approach to life, it also buttressed a central tenet of the standard theory of evolution by claiming that ‘The Central Dogma is a modern version of the Weismann Barrier’ (Noble, 2018). Dawkins went even further to formulate a central dogma of embryology:

If Crick's central dogma states that protein may not be translated back into DNA, the central dogma of embryology states that bodily form and behaviour may not be translated back into protein. (Dawkins, 1982)

These shortcuts by neo‐Darwinist biologists have further confused the situation and are not valid for the same reason as the original Central Dogma: they ignore multiple levels of function.

**FIGURE 1:**
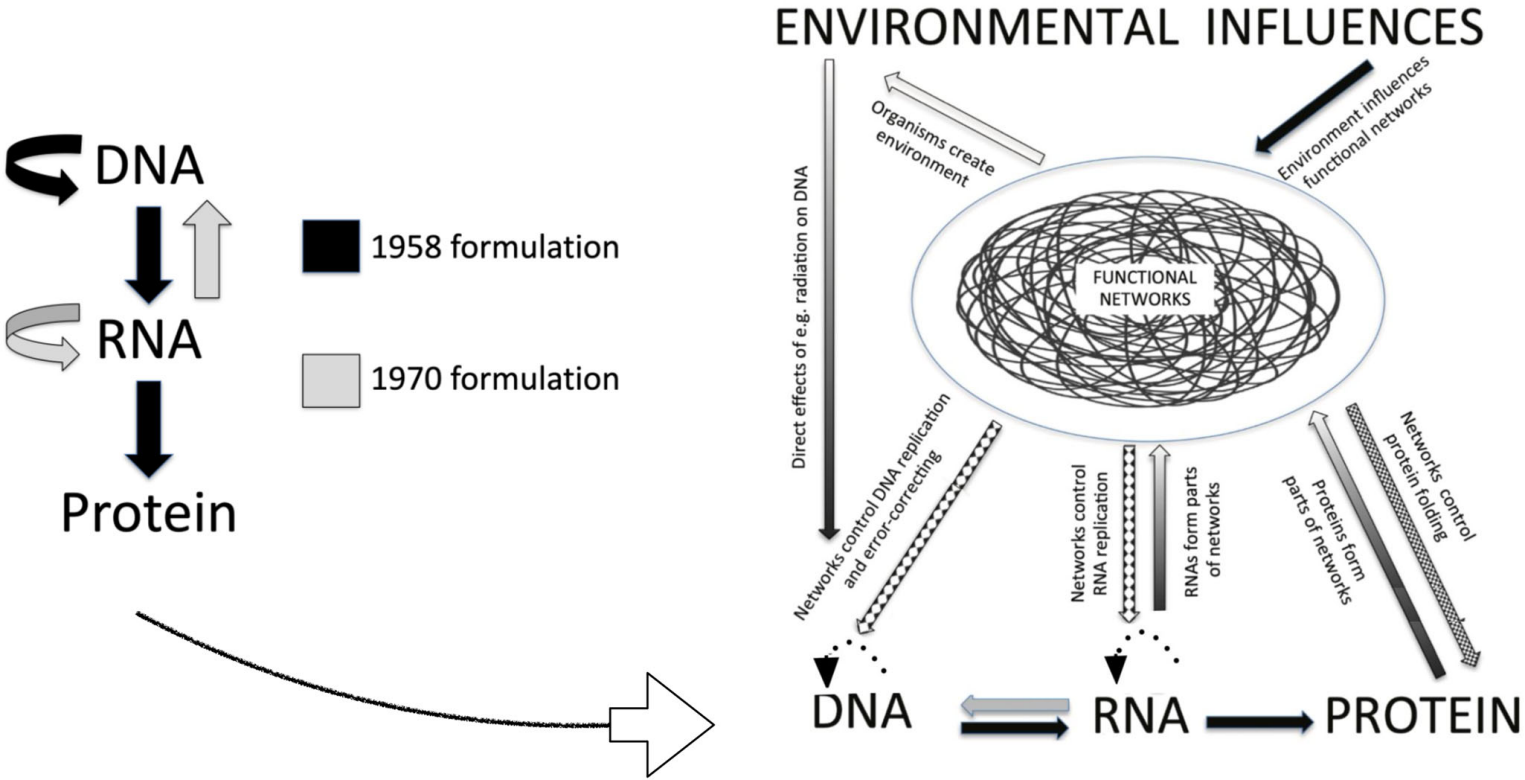
Left: Crick's 1970 revised formulation of his 1958 Central Dogma. Note that the curved self‐replication of DNA and RNA is represented by solid arrows. Right: The role of physiological functional networks in the control of DNA, RNAs and proteins (Noble, 2026). Slightly redrawn from Noble & Noble (2025, Figure 1). The dotted self‐replication arrows, indicating that self‐replication is inaccurate, have been placed above the nucleotides instead of below. The downward control arrows from the regulatory networks are then correctly placed in relation to self‐replication. Those controls are responsible for passing highly accurate copies of DNA to the daughter cells during cell division.

Crick's 1970 Central Dogma is shown on the left in Figure 1. The self‐replication arrows are solid, implying, following Schrödinger (1943), that self‐replication is robust and accurate. The Central Dogma and its neo‐Darwinist version of evolution *requires* this to be true. Otherwise, the replicator cannot be separate from its vehicle, the living cell. But there is a direct test, which is to investigate its main prediction: that the nucleotide strings will accurately self‐replicate without a living cell. Deck et al. (2011) did this on RNA, while Schulman, Yorke and Winfree (2012) did so for DNA, showing that purely chemical replication would be accurate enough for small viruses, just a few tens of thousands of base pairs long. The authors describe replication as ‘robust’ or ‘efficient’ and, as chemical processes, this is true since the accuracy is only one error per few thousand base pairs. But this error rate is wholly insufficient for genomes billions of base pairs long. In a 3 billion pair genome there would be nearly a million errors. That is the first major mistake to be corrected and it is the reason why those curved arrows are dotted in the right hand panel of Figure 1. Recent studies concerning in vitro synthesis of DNA also show its limitations (Zhou et al. 2025). Nothing approaching the extreme accuracy of cellular replication has been achieved outside a living cell.

Those natural errors in self‐replication are important. The living cell does not divide until an accuracy around 1 error in 10 billion is achieved through the orchestration of a set of cut and paste enzymes creeping along the DNA threads to replace incorrect bases. Stark and Taylor (2006) state that ‘The G2 checkpoint prevents cells from entering mitosis when DNA is damaged, providing an opportunity for repair and stopping the proliferation of damaged cells.’ That process also gives the living cell control over the accuracy of replication. The immune system uses this control to enable it to change DNA sequences when it generates new shapes of immunoglobulins to neutralise viruses or bacteria (Odegard & Schatz, 2006). Organisms also use this control to hypermutate or reorganise their genomes when under stress, as McClintock (1941, 1984) first showed in discovering mobile genetic elements, for which she was awarded a Nobel Prize in 1983. Anyway, cells with damaged DNA are recycled.

Our first extension of Principle 1 therefore is that, contrary to Crick's view, it is clear that Principle 1 is *necessarily* true since *DNA on its own is not a sufficiently accurate self‐replicator*. That in turn requires top‐down causation represented by control of the G2 checkpoint. A complete bottom‐up process of causation in biology cannot therefore exist. The essential tools of DNA editing by the immune system, and in responses to environmental stress, enable functional networks in living cells to harness the stochastic variability (D. Noble, 2017a; R. Noble & D. Noble, 2017, 2018). Multiple downward processes of causation must clearly exist.

Note also that we refer to the networks as ‘functional’ rather than using the term ‘gene regulatory networks’, since functional is what systems must be.

Function has a central significance in systems physiology and in systems engineering. In engineering, systems are deliberately developed to serve particular purposes. In physiology functions have evolved. Without serving a purpose in the life of an organism, there is no reason why a function should have been selected by evolution. Twenty‐first century evolutionary biology is bringing this use of purpose in functional physiology and its evolution back as a study of biology (Corning et al., 2023; Vane‐Wright & Corning 2023)

The term ‘gene regulatory networks’ is often misunderstood to imply that genes are doing the regulation. DNA sequences are sometimes involved in functional networks, as for example, in circadian rhythm (Foster & Kreitzman, 2004; Nurse, 2021), but even in such cases, the process is variations in gene *expression*, not in genes themselves. DNA is an inert chemical. Its sequences can be read by living cells to enable RNAs to be made, which are then used to enable ribosomes to make proteins.

To these facts we need now to add the vast extent to which the function of proteins *is determined by the living cell*. As Philip Ball (2023) writes:

…alternative splicing, the way it is regulated, and the role of disorder in enabling promiscuous protein networking, together mean that the *information processing* of the cell – if we can, with caution, use that loose computational analogy – doesn't have the architecture we thought it does. What a protein *means* for its host cell is mutable with the state of the whole cell, and is thus literally absent from the sequence of the gene encoding it. (Ball 2023)

A complete closed form, bottom‐up, process of causation in biology is impossible.

## 3 PRINCIPLE 2: TRANSMISSION OF INFORMATION IS NOT ONE‐WAY

In this section we will explain the relevant arrows showing two‐way causation in Figure 1.

First, we clarify the two upward arrows. The first is from RNA to the networks since RNAs play a major role in regulating DNA. The second is the upward arrow from proteins to the networks since proteins also play a major role as enzymes, as mechanical (muscle) proteins, as structural proteins, membrane transporters or circulating proteins.

But there is no upward arrow from DNA. Readers may think there should be since, as noted already, DNA is involved in networks like the cell cycle. But it is involved only via the networks controlling gene (DNA) *expression* levels. DNA is an inactive molecule. Its sequences are passively read by other molecular systems, which is a cause by structural form, not a cause by active dynamic processes. The active molecules it enables are the RNAs and proteins. We will deal later with the different forms of physiological causation.

The downward arrows are important since they are the means by which the living organism sidesteps the mistaken idea that the Central Dogma prevents organisms from editing their genomes. They do so by natural genetic engineering (Shapiro, 2021), *not* by reversing the chemical processes DNA → RNA → protein, but by selection of variations in the stochastic creation of new DNA sequences, e.g. by immune systems, or by organisms reacting to environmental stress. The functional networks also adapt flexibly to environmental conditions, and these changes have epigenetic actions on DNA and chromatin.

Biology has been genocentric for the last nine decades since Schrödinger (1943) introduced the idea that genes replicate like crystals do, and Crick formulated his Central Dogma (Crick 1958, 1970). It is now clear that the functions of a gene are not defined by the DNA sequence alone but also by how the gene is marked and controlled by chromatin modification, DNA methylation and noncoding RNAs. *Self*‐replication ‘like a crystal’ is far from accurate enough; the extreme accuracy is instead ensured by control processes in living cells.

Epigenetic control of gene expression is stable and long‐term yet reversible and responsive. The epigenome programs the genome (Szyf et al. 2008) through such harnessing of stochasticity, and there are different stochastic types (Rahman, 2008). The process becomes directional, no longer stochastic (R. Noble & D. Noble, 2018; Noble & Hunter, 2020; van Heyningen, 2024).

## 4 PRINCIPLE 3: DNA IS NOT THE SOLE TRANSMITTER OF INHERITANCE

We begin this section by noting an important difference between the differential and integral ways of assessing genetic inheritance, i.e. what characteristics of an organism a particular gene is responsible for. This difference was first pointed out by one of us in 2008 in an article on ‘Genes and Causation’ (Noble, 2008b):

…differences cannot reveal the totality of functions that a gene may be involved in, since they cannot reveal all the effects that are common to the wild and mutated types. We may be looking at the tip of an iceberg. And we may even be looking at the wrong tip since we may be identifying a gene through the pathological effects of just one of its mutations rather than what it does for which it must have been selected. (Noble 2008b)

A gene bringing a pathological phenotype following one mutation ceases to be operational for the proper survival of the individual, unless there are other, perhaps hidden, beneficial phenotypes. Those beneficial effects may not be evident until an environmental change makes them so.

Since that publication it has become even more evident that the differences between wild and mutated types cannot, by themselves, reveal functional causation since very small, and even zero, association scores (associations between genes and functions) can occur even for genes that are strongly causal. That was shown in work on the heart sinus node pacemaker by Noble et al. (1992) (see also Noble, 2026). Blocking a protein whose template is an HCN1 gene shows only a 10–15% association score, whereas the action of its protein in generating the electrical current for the pacemaker depolarisation was measured as up to 85% (Noble, 2026). These studies reveal the limitations of association scores since they pin the causation down in experimental findings. Moreover, the large difference between association and causation depends on the degree of genetic buffering, and on many other non‐genetic factors determining the difference between association and causation. In the extreme case, a gene variant showing even a zero association score may in fact be strongly causal. Functions essential for life are robust, with alternative networks. The larger the buffering, the smaller the association.

The genetic buffering capacity depends on the environment of other genes since they all act as templates Mistaking association for causation for RNAs and proteins forming functional networks, together with metabolic and hormonal chemicals. It must also depend on the environment. That was revealed in the study of Hillenmeyer et al. (2008) in yeast. Of the 6000 genes, they showed that 80% show a zero association score (meaning no effect at all on metabolism or reproduction) in ideal nutritional environments. But, once key nutrients are lacking in the environment, large association scores appear for the great majority that were previously silent.

Physiology is the study of causation. Genomics is the study of association. They need to be combined for a complete understanding of living organisms.

### 4.1 The gene‐centric impasse in healthcare

The problem created by mistaking association for causation is now acute for the medical sciences since it creates a gene‐centric impasse. Around 90% of associations in GWAS are very small, except for rare mono‐genetic diseases affecting 5% of the population. Small associations are more likely a sign that causation is hidden within robustness. Nor does adding association scores together to produce polygenic scores solve the problem. They are fictional, as already noted, but their greatest failure is that they do not reliably predict major fatal diseases (Hingorani et al. 2023).

We will return to this impasse later, when we address the question ‘how physiology can solve the gene‐centric impasse?’

### 4.2 Non‐DNA inheritance

So far we have shown that DNA inheritance cannot be the complete story. Both DNA and cytoplasm contribute to global inheritance. This is also observed in assisted reproductive technology (ART) where embryos exhibit adaptability to the environment thus revealing phenotypic plasticity (Charney, 2013). Principle 3 goes much further than the difference between association and causation since it states that DNA is not the sole transmitter of inheritance. Here too there have been many advances.

Importantly, there are no genes ‘for’ lipid molecules in tightly packed membranes. Genes, as DNA, only template for RNAs and proteins. Furthermore, membranes can self‐template. Phospholipids automatically form and grow membranes. The self‐assembly of biomolecule mixtures, which could be used for designing lipid molecule‐based bio‐membranes in solutions, has been intensively studied (Sun, Pan & Li, 2022).

Does it matter, or are all egg cells much the same? Sun et al. (2005) cloned fish using a fertilised, but enucleated, egg cell from a goldfish and a nucleus from a carp. If the nuclear DNA determines everything, the resulting organism should be a carp. Instead it had a vertebra number around half way between a carp and a goldfish. The title of their article includes the reason: ‘Cytoplasmic impact on cross‐genus cloned fish…’. This is not an isolated example, but certainly one of the most striking ones. Physiologists have documented maternal and paternal transmission of characteristics (Gluckman & Hansen, 2006, 2011; Camus et al., 2022) and we now know that extracellular vesicles containing, for example, metabolic information in their RNAs, can pass to the germ cell line, thus crossing the Weismann Barrier. In fact, many such signals communicate to the germline (Smith & Spadafora, 2005; Spadafora 2023; see review by Phillips & Noble, 2024).

### 4.3 PRINCIPLE 4: THE THEORY OF BIOLOGICAL RELATIVITY: THERE IS NO PRIVILEGED LEVEL OF CAUSALITY

This is the main principle of the set of eight, from which all the others can be derived. The first full article on the principle was published as ‘A Theory of Biological Relativity’ in a Special Issue of *Interface Focu*s (Noble, 2012) and has now received more than 500 citations in fields as widespread as philosophy, physics, mathematics, engineering, physiology, biology, evolutionary biology, semantics, economics and sociology. It also became the subtitle of the book *Dance to The Tune of Life* (Noble, 2016). The idea is best described as a principle, within which specific theories can be developed.

### 4.4 Relation with other principles of relativity

First, we clarify the relationship to principles and theories of relativity in physics. That begins with the *General Principle of Relativity* (Wald, 1984; Carroll, 2004), which was already implicit in Newton's action and reaction idea, as it was also in East Asian Taoist and Buddhist philosophies: that nothing exists in and of itself; and in the work of Heraclitus, a pre‐socratic philosopher of being as process, not a thing. The modern western philosophers who championed this idea are the mathematician Alfred North Whitehead (Whitehead, 1929) and the philosopher John Dupré (Dupré, 2012; Nicholson & Dupré, 2018).

The idea is simply that something that did is supposed to not interact with anything else would never even be observable, a precondition for existing in any meaningful sense. Observation therefore already involves two‐way interaction, as the revolution that led to Quantum Mechanics made clear. That is also the meaning of the no‐self idea in Eastern Philosophy. In physics it became encapsulated in the requirement that the equations describing laws should have the same form in all frames of reference.

Einstein's theory of General Relativity is an interaction between objects with mass and the dimensions of space‐time in which they move. Gravity, conceived of as a force, is replaced by the curvature of space‐time. Objects and waves then move through curved space‐time. It is therefore also a theory of two‐way interaction. The masses create a form, space‐time, which constrains how objects and waves naturally move within it. It is therefore also a theory of the relativity of causation. Physics and biology are different, but physics underlies biology (Ellis & Kopel, 2019).

Biological Relativity is the same causal principle applied to levels of organisation in biological systems, which constrain the movements of objects within them: the movements of molecules in biological systems are constrained by the boundaries (cell membrane) and internal structures (mitochondria, endoplasmic reticulum, ribosomes, nucleus, etc.) of the cell. Each level of organisation (tissues, organs, systems, social systems) then acts as a set of boundary conditions within which the differential equations of motion of the lower level components must operate. The idea that there is a fundamental difference between causation by form (passive causation) and efficient causation (by movement) was first enunciated by the philosopher and scientist Aristotle more than 2000 years ago.

### 4.5 Relativity is a necessity not an option

One of the reasons why ‘principle’ is a better description of biological relativity than ‘theory’ is that, as a principle, it is mathematically necessary, whereas theories must be falsifiable conjectures. The necessity can be seen by considering how any mathematical model of a biological system must be constructed.

Consider what happened when a set of auto‐catalytic reactions (Kaufman, 1993, 1995) first became enclosed in a cell membrane. We don't know when that may have happened, how many times it happened before it ‘succeeded’, nor where it happened, but once it happened biological relativity began!

First, instead of the set naturally dispersing through diffusion, so ceasing to exist as a set, it would interact indefinitely. It is easy to envisage this happening. As Bangham and Horne (1964) first showed, merely dropping lipid molecules into water automatically forms liposomes, bubble‐like droplets with double‐lipid membrane. Now, more complex biomembranes are synthesised with conventional phospholipids, synthetic polymers, nanoparticles and a cytoskeleton‐like structure (Yasuhara & Morigaki, 2022).

Second, once that has happened a set of new global parameters arise. Depending on the molecules present, and the permeability of the membranes, those liposomes would automatically generate electrical potentials, pH, concentrations of the ions of various salts and other molecules, just as living cells do today. The mere existence of a cell membrane ensures that these properties will arise. Notice, though, that they are not themselves individual molecules. Like temperature, they are properties of *systems* of individual molecules. They are the equivalent of thermodynamic parameters. That creates feedback interactions between individual molecules and the properties of the system. One of the most important of these in cell physiology is the Hodgkin cycle.

### 4.6 The Hodgkin cycle as an origin of the principle of biological relativity

The Hodgkin cycle underlies Hodgkin and Huxley's (1952) model of the nerve impulse describing the feedback loop between the cell voltage (a global property) and the generation of electric current by ion channels.

**FIGURE 2:**
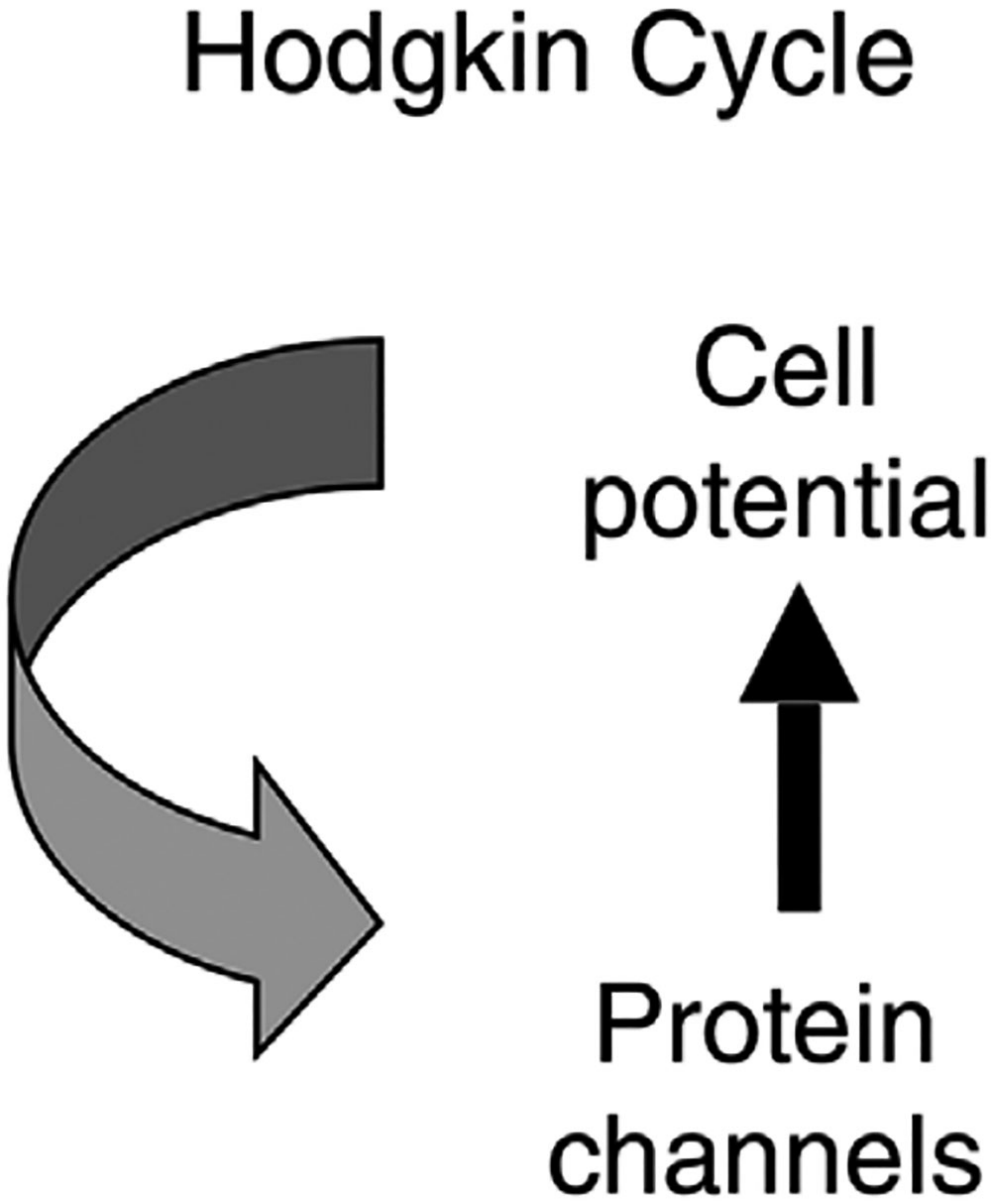
The Hodgkin cycle. Protein channels generate flow of ions, so creating the cell electrical potential, which in turn controls the opening and closing of the channels.

The influence of the cell electrical potential on the ion channels is described by differential equations corresponding to the opening and closing of the gates that determine the electric current. Those charge movements then get summed together to form the electrical potential across the membrane, i.e. the membrane also acts as a capacitor. It is therefore a cycle between two levels of organisation: the cell as a whole and the opening and closing movements of individual ion channels (Figure 2).

**FIGURE 3:**
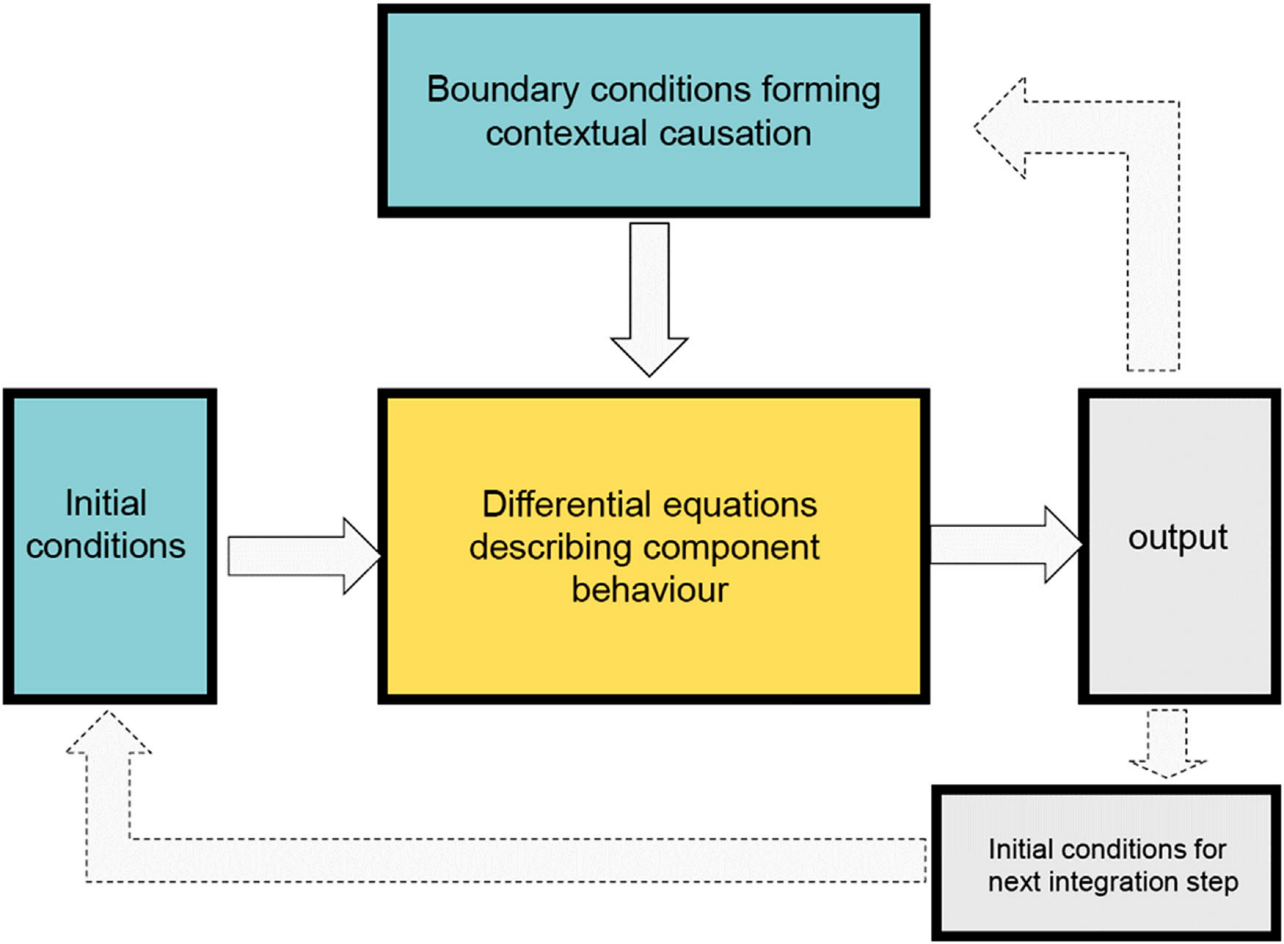
Left: the stages of an integration process. The centre (yellow) is the dynamic differential equations describing the kinetics of how the molecules forming ion channels react to changes in the initial and boundary conditions (blue‐green boxes). To produce an output (grey) both the conditions and the kinetics need to be solved by the integrator. That output is then used to form the initial conditions for the next time step of the integration. Right: the boundary conditions are global properties of the upper level (the cell). They form downward causation, whereas the differential equations form upward causation. Upward and downward here are metaphors. In reality, the boundary conditions, in particular, surround rather than sit on top of the molecular events.

This kind of cycle between two levels of organisation should be clearly distinguished from metabolic cycles, such as the citric acid/Krebs cycle. That describes a cycle of *sequential* reactions, as distinct from simultaneous ones, between the various stages of enzyme activity, whereas the Hodgkin cycle describes a *simultaneous* interaction between two levels of organisation, cellular and molecular. That is represented in Figure 3, showing how the differential equations are integrated, and why the integration of initial and boundary conditions with the kinetic molecular movements are simultaneous.

The Hodgkin cycle is a cell/molecular cycle. But similar cycles must exist between all levels of organisation in a living organism. Tissues organise cells, organs organise tissues, systems organise organs and whole organism processes, while the organism itself interacts with its environment both physically and socially. The right hand part of Figure 3 therefore represents the equivalents of Hodgkin cycles between each pair of levels. There is a meshing of causation between each pair of levels. There can be different mechanisms underlying a cycle or pathway and pathways can be described without knowing the mechanisms creating them (Ross, 2021).

Of course, simultaneous Hodgkin‐like cycles can combine with sequential metabolic cycles, as they must do in mitochondria, which do not work to produce ATP when depolarised. The relationship is complicated since mild depolarisation is a component of anti‐ageing processes (Vyssokikh, et al. 2020).

**FIGURE 4:**
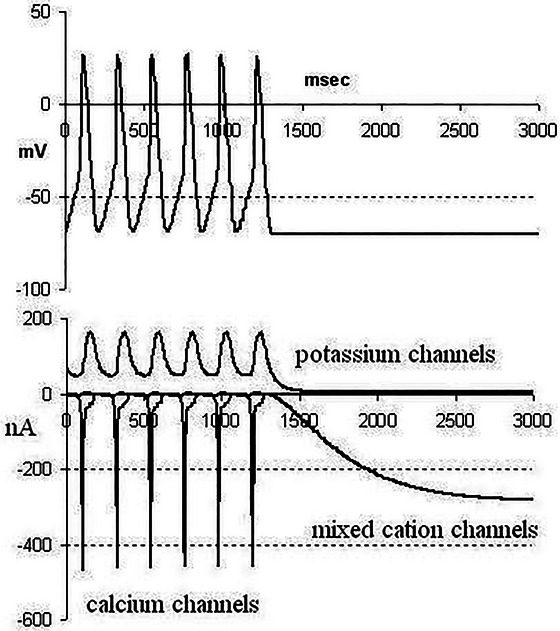
Illustration of the necessity of boundary conditions in biological relativity. An experimentally based model (D. Noble & S.J. Noble, 1984) is used to show that while the cell voltage is allowed to vary freely as electric current flows, the electrical rhythm continues both at the cell (electrical) level and in the activity of the molecular ion channel proteins. But as soon as the boundary condition (the cell voltage in this example) is held constant the ion channel rhythms also cease.

We can now explain why we wrote that the principle of biological relativity is a mathematical necessity. Figure 4 shows how essential the Hodgkin cycle is to rhythmic activity in the heart. As soon as the boundary condition (the cell voltage in this example) is held constant the ion channel rhythms also cease.

### 4.7 The harnessing of stochasticity

All of these processes are subject to another important property of living organisms. All organisms are based on solutions and suspensions of complex molecules in water. Water is a very unusual solvent. It is liquid at terrestrial temperatures, as well as occurring as solid ice and as a steamy gas, whereas its constituent atoms would be gases way below terrestrial temperatures. Unusually also, the molecules in ice move apart and become lighter than liquid water. Ice therefore forms at the surface of ponds and lakes. When the earth may have been completely covered like an ice ball (Alderman & Tilley, 1960), life continued in the liquid below the ice at around 4°C, which is the temperature at which water reaches its maximal density.

Liquid water is a huge source of molecular‐level stochasticity, first shown by Robert Brown (1828). He viewed fine pollen grains sprinkled on the surface of water under a microscope. The particles were observed to continually move around stochastically, sometimes moving long distances. Einstein showed in 1905 that this was due to water molecules continually bombarding the suspended particles. Water molecules are not as free inside living cells since many form hydration shells around the macromolecules. Even if a fraction of cellular water has slower dynamics, the global water dynamics are still similar to that of pure water (Lang et al. 2024), while the slow water is fine‐tuning the protein environment and is evolutionarily adaptive (Ball, 2017). The relatively free water molecules still buffet the cell's molecules, causing them to move around.

Even the smallest living organism, a bacterium around 1 by 4 µm, contains around 20 billion water molecules. In addition to the breaks in macromolecules like DNA caused by radiation there will be breaks caused by this stochastic behaviour. Those breaks will contribute to the observed chemical fact that the error rate in DNA self‐replication is too high for accurate copies of a cell's DNA to occur before it divides. As we have already noted on Principle 1, this is what gives living cells control over how they use stochastic variations in DNA. Stochasticity is also involved at higher levels including the individual organism level in its behaviour (Honegger & de Bivort, 2018)

Physiological examples of the harnessing of stochasticity can be found in the immune system which chooses successful immunoglobulins out of millions generated by stochastic hypermutation in its B and T cells (Odegard & Schatz, 2006), and in the nervous system where a form of neural selection occurs (Edelman, 1987, 2012).

The principle of biological relativity therefore includes this use of harnessed stochasticity between all levels of organisation. Each level accesses the stochasticity in the level below.

#### 4.7.1 The openness of living systems

**FIGURE 5:**
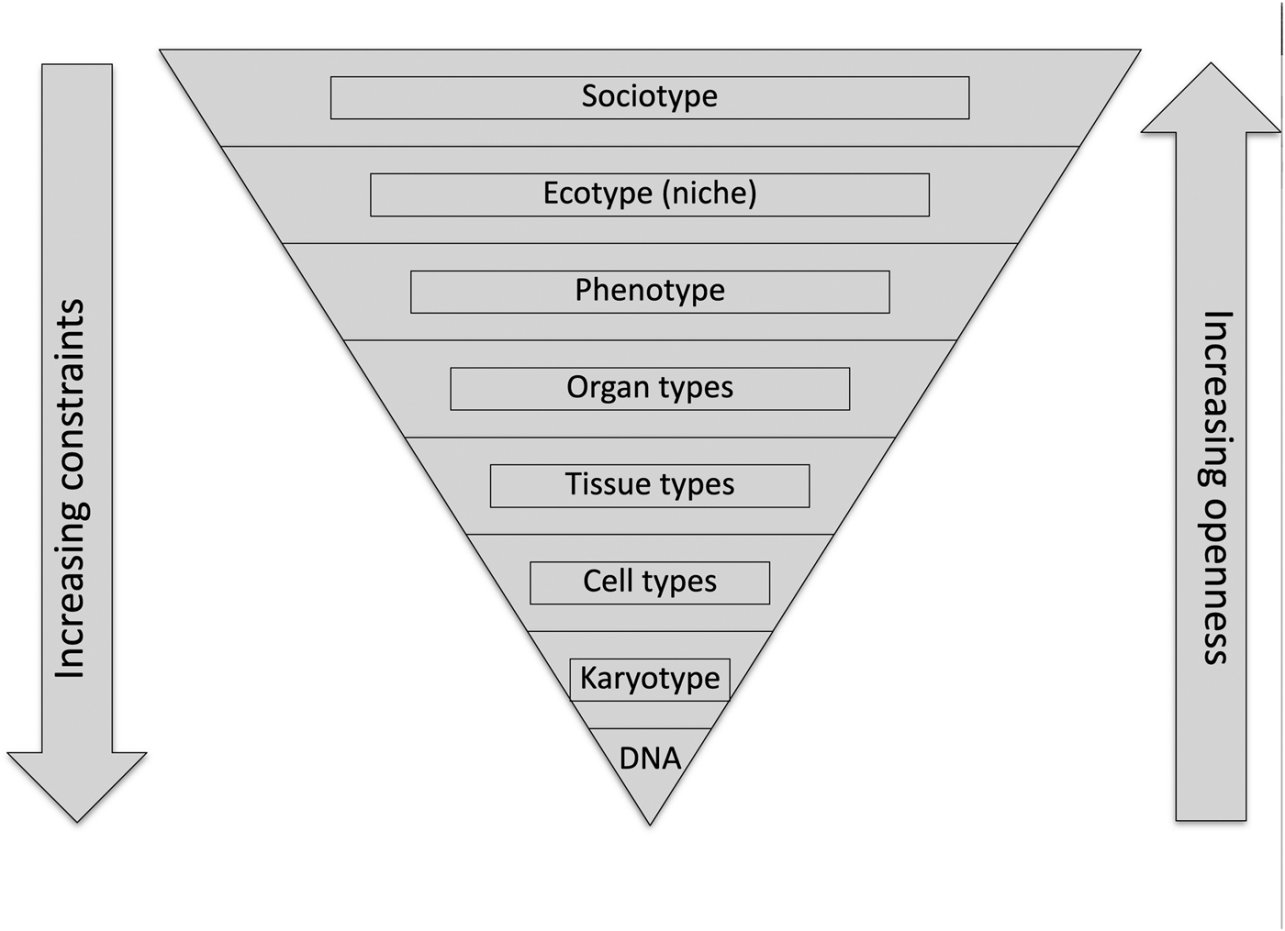
Levels of organisation in living systems. The most constrained will be the lowest (molecular) levels. The most open will be the organism as a whole (phenotype) interacting with the ecotype and the sociotype. As we pass downwards towards DNA, the levels become increasingly constrained. By contrast, proceeding upwards the levels become increasingly open. (From Noble & Noble, 2023)

One of the consequences of the way in which each level of organisation can harness the stochasticity of lower levels is that organisms cannot be closed systems. In such a meshing of levels of organisation, the most constrained level will be the lowest level, subject to all the constraints of the higher levels. The most open will be the social interactions with the organism as a whole. This is illustrated in Figure 5.

## 5 PRINCIPLE 5: GENE ONTOLOGY WILL FAIL WITHOUT HIGHER‐LEVEL INSIGHTS

The main point is obvious. The names we use for genes depend on the way in which the first functionality of any particular gene was identified. But there may be hundreds of other functionalities. The naming of a gene is the privilege of those who first discovered a function.

A Gene Ontology will naturally reflect this. Provided that we all bear this in mind, no‐one should be confused. This statement was included in the set of principles in the 2008 article because the tendency to think in terms of simple relations between genes and functions (Beadle & Tatum, 1941) dies hard. We see the discovery of multiple functions of genes as an important part of a systems view of biology. Naming genes with single functions confuses this issue.

## 6 PRINCIPLE 6: THERE IS NO GENETIC PROGRAM

The main development since 2008 is the introduction of the harnessing of stochasticity (Noble, 2017b; Noble & Noble, 2018, 2020). This concept could even qualify for a statement of principle on its own. It is a necessary feature of living systems based on water as the solvent, since that automatically generates massive stochastic variation. As we have already shown with examples, higher levels can harness this for selected forms of functions and behaviour among many variations. In opposition to the idea that DNA is a blueprint or programme for life, DNA is really just a database. DNA does not play an active role in any of the functional networks. Its products, RNAs and proteins, do that.

## 7 PRINCIPLE 7: THERE ARE NO PROGRAMS AT ANY OTHER LEVEL

‘If we can't find the program of life at the level of the genome, at what level do we find it? The answer is “nowhere!”’ (Noble, 2008a, p. 24)

The 2008 article clarifies this enigmatic statement by showing that any program we may identify in a living system simply *is* the functionality itself in the organism, which is necessarily true in open systems. There is therefore no program separate from the functionality itself. That openness includes the social environment, which cannot be fully predicted. There is stochasticity, therefore, not only at levels below each organisational level within the organism, stochasticity also exists in social interactions. Stochasticity exists at the elementary molecular level and at the abstract level of social interactions (Honegger & de Bivort, 2018). Living organisms can be predictable to the extent that they use well‐rehearsed responses to anticipate many challenges, but we can never exclude the influence of the living agent itself. A conscious action is more a commitment than a physically determinable act.

## 8 PRINCIPLE 8: THERE ARE NO PROGRAMS IN THE BRAIN

Many neuroscientists use the analogy of the functions of the brain being comparable to the programming of computer systems, leading to the question where in the brain all this may be controlled? Crick chose to highlight the claustrum as the location of consciousness, but recent research has indicated that it rather plays a role in attention and sleep/wakefulness and works more as an interface: ‘it is perhaps not the “seat‐of‐consciousness”’ (Smith et al. 2020) – the claustrum is an interface implied in attention and slow wave sleep but it is not shown to be the seat of consciousness.

These last three principles can now be subsumed into a combined principle on the program idea. The genome is a database, not itself a program. What we may be tempted to call ‘programs’ in open living systems is redundant since any such programs are themselves the functionality (Coen, 1999).

## 9 CONCLUSIONS

In the light of the many substantial developments in response to the original 2008 article, the principles of systems biology can now be formulated in rigorous mathematical engineering terms. An important conclusion from these developments is that the main principle, that of the principle of biological relativity, is a logical necessity. No valid approach to understanding living organisms can any longer ignore higher levels of organisation, since no system can exist without the form of the system itself, which then forms the boundary conditions through which all the lower processes can be integrated. This conclusion is a logical necessity.

A second important conclusion is that the alternative, reductionist approach to understanding living systems, based on the original Central Dogma of molecular biology, has comprehensively failed to deliver the hoped‐for benefits in health care. Interpreting functions and diseases purely from genomics has in practice been a failure. It is time for physiology to demonstrate the way back to systems approaches that can successfully address the gene‐centric impasse.

## AUTHOR CONTRIBUTIONS

Both authors contributed to all stages in the writing of this article. Both authors have read and approved the final version of this manuscript and agree to be accountable for all aspects of the work in ensuring that questions related to the accuracy or integrity of any part of the work are appropriately investigated and resolved. All persons designated as authors qualify for authorship, and all those who qualify for authorship are listed.

## CONFLICT OF INTEREST

None declared.

## FUNDING INFORMATION

None.
